# Formalin injection causes a coordinated spinal cord CO/NO-cGMP signaling system response

**DOI:** 10.1186/1744-8069-1-33

**Published:** 2005-11-18

**Authors:** Xiaoyou Shi, Xiangqi Li, J David Clark

**Affiliations:** 1 Stanford University Department of Anesthesiology, Stanford, CA, USA; 2 Veterans Affairs Palo Alto Healthcare System, Palo Alto, CA, USA

## Abstract

**Background:**

The CO/NO-cGMP signalling system participates in the regulation of many physiological processes. The roles this system plays in spinal cord nociceptive signalling are particularly important. While individual components have been examined in isolation, little study has been dedicated to understanding the regulation and functioning of the system as a whole.

**Results:**

In these studies we examined the time course of expression of 13 genes coding for components of this system including isoforms of the heme oxygenase (HO), nitric oxide synthase (NOS), soluble guanylate cyclase (sGC), cGMP dependent protein kinase (PKG) and phosphodiesterase (PDE) enzyme systems. Of the 13 genes studied, 11 had spinal cord mRNA levels elevated at one or more time points up to 48 hours after hindpaw formalin injection. Of the 11 with elevated mRNA, 8 had elevated protein levels 48 hours after formalin injection when mechanical allodynia was maximal. No component had an increased protein level which did not have an increased mRNA level at one or more time points. Injection of morphine 10 mg/kg prior to formalin completely abolished the acute nociceptive behaviours, but did not alter the degree of sensitivity which developed in the formalin treated hind paws during the subsequent 48 hours. Morphine treatment did, however, eliminate formalin induced increases in enzyme protein levels.

**Conclusion:**

Our results indicate that the expression of the components of the CO/NO-cGMP signalling system seems to be coordinated in such a way that a generalized multi-level enhancement rather than a tightly limited step specific response occurs with noxious stimulation. Furthermore, the analgesic morphine administered prior to noxious stimulation can prevent long-term changes in gene expression though not necessarily nociceptive sensitisation.

## Background

Traditional approaches to the study of nociceptive signaling mechanisms often focus on single gene products. The functions of such molecules are commonly examined using pharmacological, electrophysiological, genetic and behavioural techniques. While there is little doubt that these investigations have been of substantial utility in understanding nociceptive signaling, it is widely appreciated that nociceptive signaling involves systems of interacting component molecules rather than individual molecules functioning independently. Furthermore, experimental paradigms are often optimised to highlight the roles of the molecules of interest making it difficult to integrate the existing literature towards a better understanding of how signaling system components function together under a single well defined set of parameters.

The analysis of changes in gene expression in models of pain is one type of paradigm often used to infer participation of a gene product in nociceptive signaling. Unfortunately, examination of the existing literature reveals the use of different species, strains, types of noxious stimulation, tissues analysed, time courses, mRNA versus protein measurement and other methods of analysis. Few studies have carefully examined changes in gene expression for large sets of genes coding for the many members of nociception-related signaling pathways.

The spinal cord CO/NO-cGMP signaling system is one of the best studied nociceptive signaling systems, and the available reports demonstrate some of the issues introduced above. The monoxides CO and NO are produced by heme oxygenase (HO) and nitric oxide synthase (NOS) respectively. The roles of HO in many pain models have been examined with changes in the expression of the spinal cord HO-2 isoform reported for both neuropathic and incisional pain models, though always studied apart from other CO/NO-cGMP signaling system components [1-4]. Likewise for NOS, increases in the spinal cord expression of nNOS I, iNOS and eNOS have been reported after noxious stimulation of various types. In 2 reports 2 NOS species were studied simultaneously after a noxious inflammatory stimulus with Wu et al. reporting increased spinal cord expression of nNOS and iNOS after capsaicin injection [5], but Tao et al. reporting increased expression of nNOS but not iNOS after carrageenan injection [6]. Once produced, these monoxides converge on guanylate cyclase to stimulate the production of cGMP. For soluble guanylate cyclase, type 1α (sGC1α), up-regulation in a model of inflammatory pain has been described, though other isoforms are not well studied [7]. The presumed target of cGMP is cGMP dependent protein kinase (PKG). PKG1α undergoes up-regulation in spinal cord tissue though PKG1β and PKG II expression do not appear to have been studied [8,9]. Finally, various phosphodiesterases (PDE's) can metabolise cGMP thus terminating its signaling functions, but we could identify no studies following the spinal cord expression of these enzymes in any model of pain. Basal expression of PDE2,3 and 5 has been demonstrated in spinal cord tissue [10,11].

Thus while the data pertaining to gene expression for some CO/NO-cGMP signaling system components and the large number of pharmacological studies not cited above support roles for this system in nociceptive signal transmission, few studies have attempted to follow the expression of the various components under standardized conditions. Such data would be useful in understanding how the system may be regulated as a whole in the setting of a specific type of pain. When 10 components of the spinal cord CO/NO-cGMP signaling system were studied together during the chronic exposure of mice to morphine, it was observed that 7 of the components were up-regulated in a coordinated fashion [12], thus coordination of expression in a nociceptive system seems plausible. The studies outlined below follow the expression of a set of 13 genes coding for the various components of the spinal cord CO/NO-cGMP signaling system over time in the formalin model of inflammatory pain.

## Results

### Formalin testing

**Figure 1:**
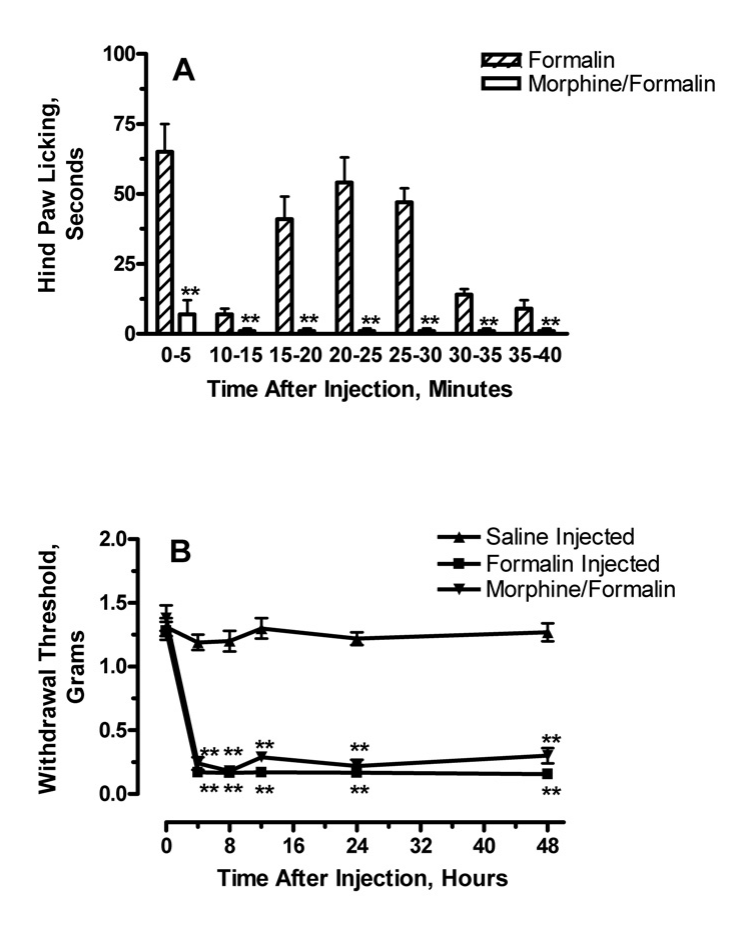
Nociceptive behaviours displayed after formalin injection. In panel A the nociceptive behaviour of mice is presented as the total number of seconds spent licking in 5 minute intervals beginning with the injection of formalin. Some mice were pre-treated with a 10 mg/kg injection of morphine. In panel B, the time course of formalin-induced mechanical allodynia with and without morphine pre-treatment is displayed. Mechanical withdrawal thresholds were followed using von Frey fibers. Data are presented as mean licking times or thresholds +/- SEM, **p < 0.01.

To characterize the spontaneous pain behaviors and persistent mechanical allodynia associated with the hindpaw injection of formalin in the C57Bl/6J mice used in subsequent experiments, we first measured the total times spent licking the injected hind paws after formalin injection. The phase I (0–5 min) average licking behavior was 65 +/- 10 sec, and the total phase II (10–40 min) average was 179 +/- 20 sec similar to the observations reported earlier by our laboratory [2] (Figure 1A). The administration of 10 mg/kg morphine prior to formalin injection nearly completely eliminated formalin-induced licking behaviors during phase I and phase II.

In Figure 1B data from experiments in which mechanical von Frey withdrawal thresholds were measured in mice up to 48 hours after formalin injection are presented. This mechanical nociceptive sensitization is a more chronic consequence of formalin injection than the phase I and II licking behavior. The mechanical thresholds in the formalin injected mice were significantly lower than those observed in saline-injected animals at all time points after formalin injection (P < 0.01), and the allodynia was relatively stable over this 48 hour time course. The administration of morphine did not significantly alter the measured allodynia over this time course.

### Expression of components of the CO/NO-cGMP signaling system – mRNA

**Figure 2:**
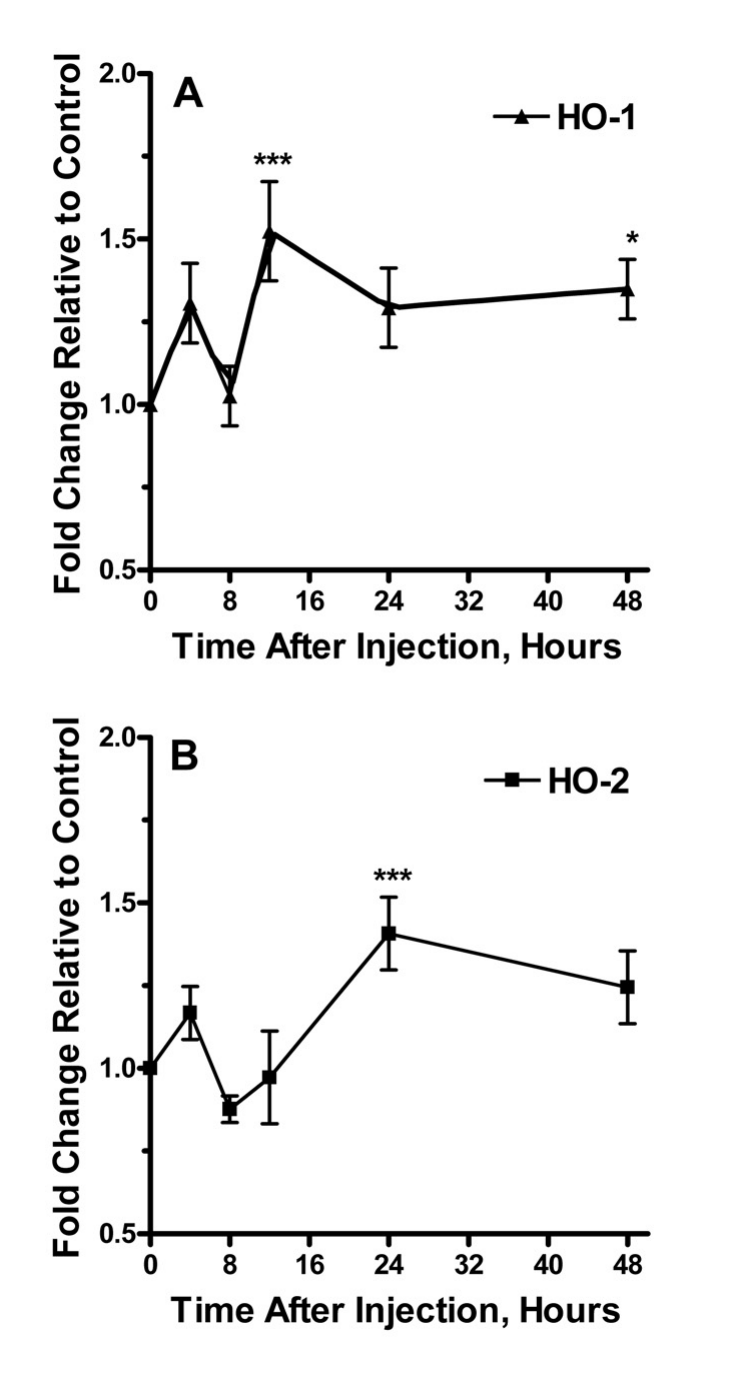
Alterations in expression of HO isoforms after the hind paw injection of formalin. Panel A provides a time course analysis of changes in levels of HO-1 mRNA up to 48 hours after formalin injection relative to controls. Panel B summarizes measurements for HO-2 mRNA. Data are presented as mean values +/- S.E.M, *p < 0.05, **p < 0.01, ***p < 0.001.

**Figure 3:**
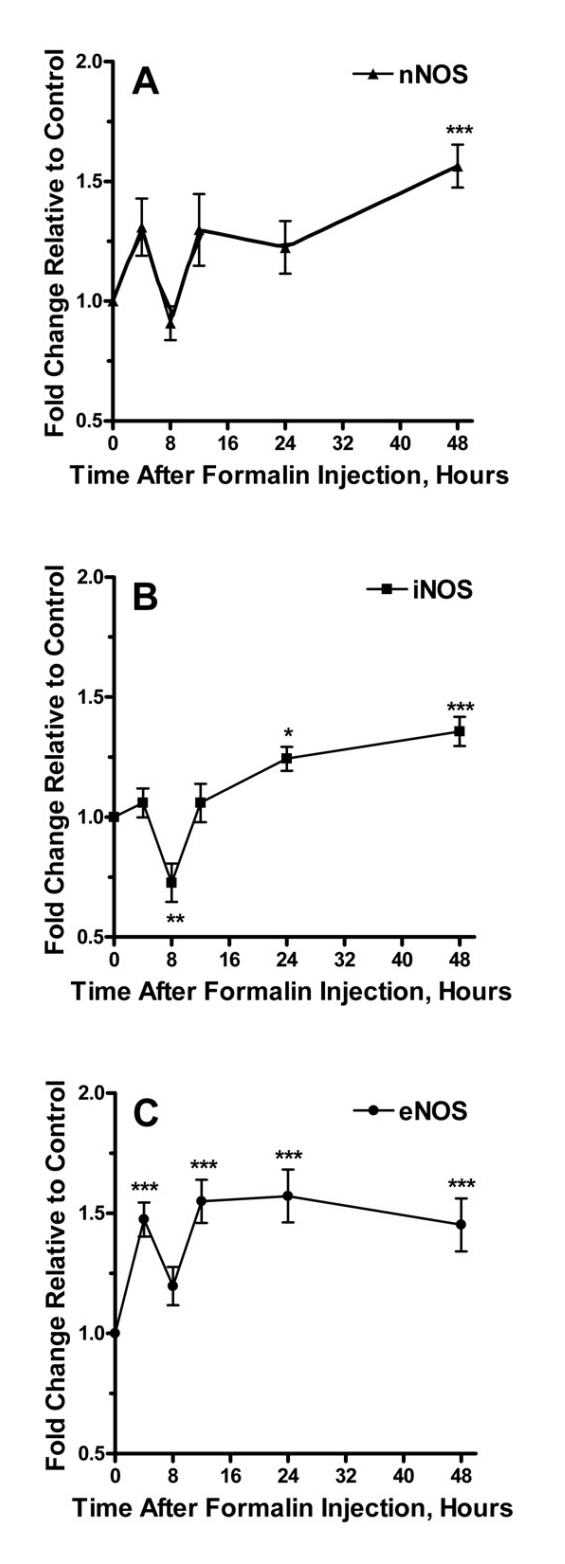
Alterations in expression of NOS isoforms after the hind paw injection of formalin. Panel A provides a time course analysis of changes in levels of nNOS mRNA up to 48 hours after formalin injection relative to controls. Panels B and C summarize measurements for iNOS and eNOS mRNA respectively. Data are presented as mean values +/- S.E.M, *p < 0.05, **p < 0.01, ***p < 0.001.

**Figure 4:**
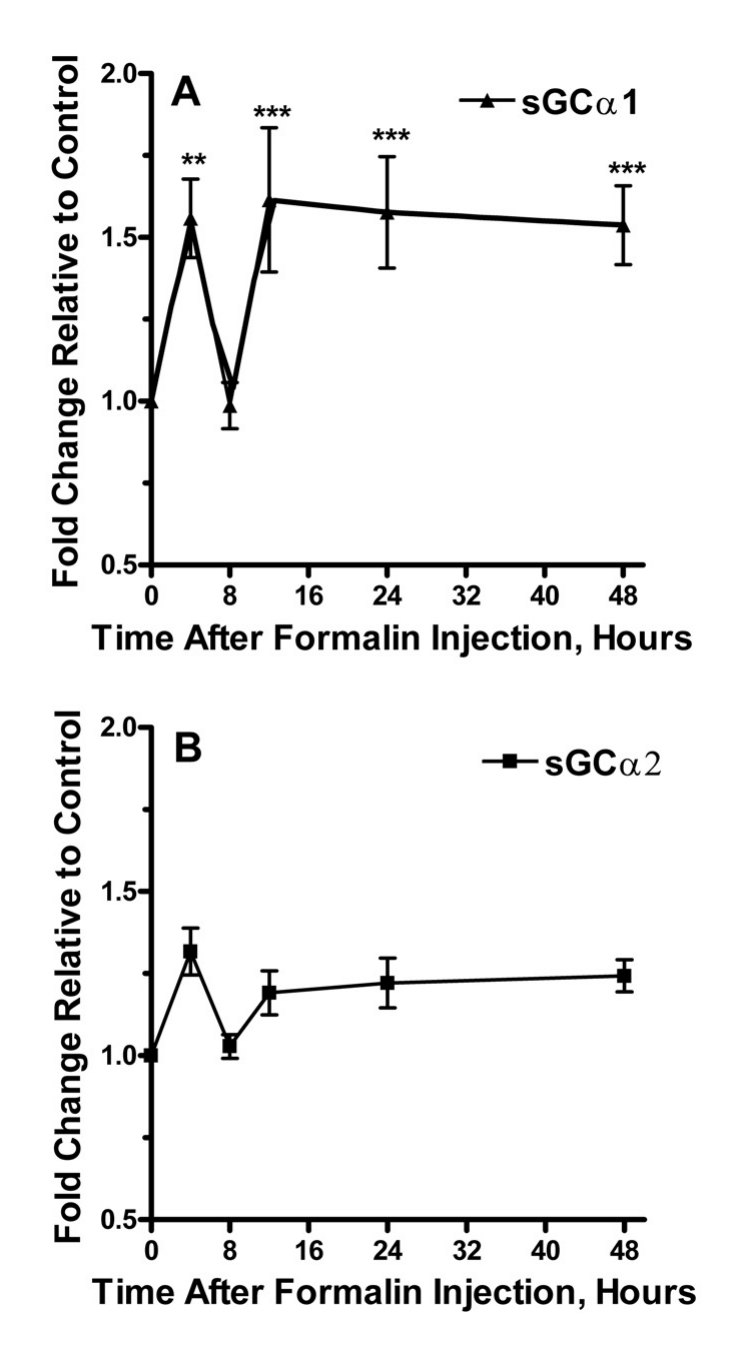
Alterations in expression of sGC isoforms after the hind paw injection of formalin. Panel A provides a time course analysis of changes in levels of sGCα1 mRNA up to 48 hours after formalin injection relative to controls. Panel B summarizes these measurements for sGCα2 mRNA. Data are presented as mean values +/- S.E.M, *p < 0.05, **p < 0.01, ***p < 0.001.

**Figure 5:**
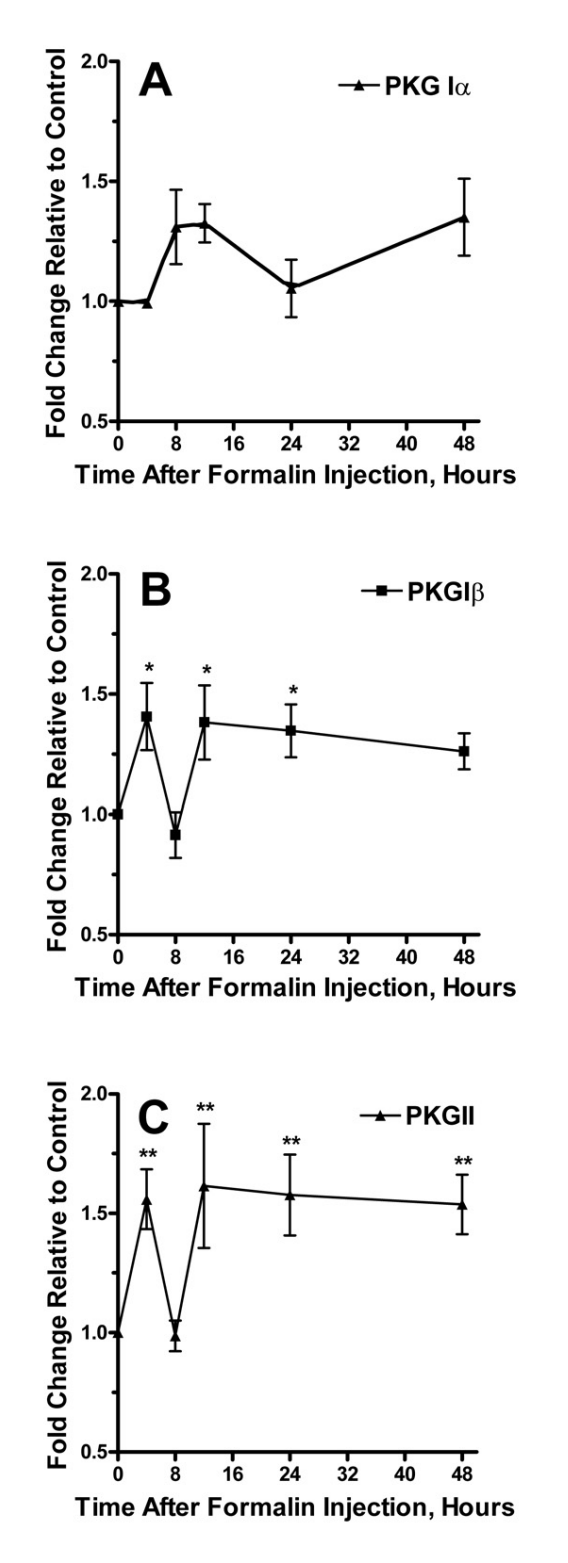
Alterations in expression of PKG isoforms after the hind paw injection of formalin. Panel A provides a time course analysis of changes in levels of PKG1α mRNA up to 48 hours after formalin injection relative to controls. Panels B and C summarize measurements for PKG 1β and PKG II mRNA respectively. Data are presented as mean values +/- S.E.M, *p < 0.05, **p < 0.01, ***p < 0.001.

**Figure 6:**
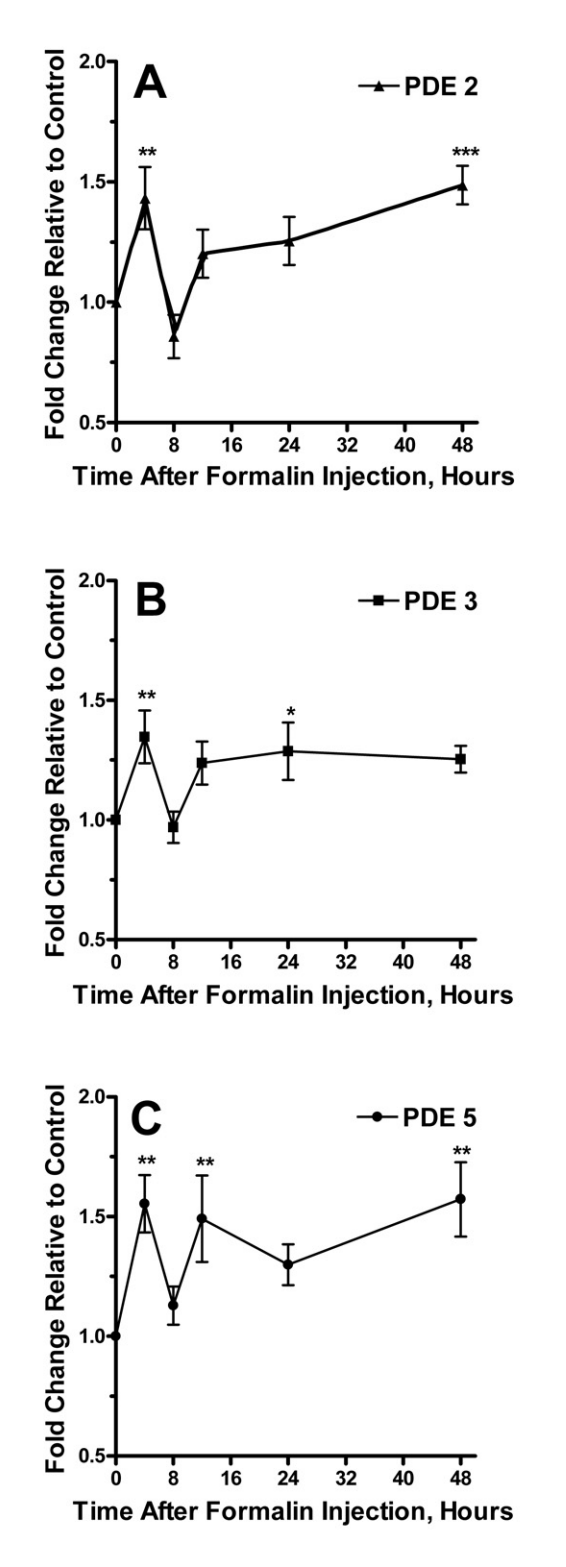
Alterations in expression of PDE isoforms after the hind paw injection of formalin. Panel A provides a time course analysis of changes in levels of PDE 2 mRNA up to 48 hours after formalin injection relative to controls. Panels B and C summarize measurements for PDE 3 and PDE 5 mRNA respectively. Data are presented as mean values +/- S.E.M, *p < 0.05, **p < 0.01, ***p < 0.001.

To examine possible changes in expression in the various components of the CO/NO-cGMP signaling system, we harvested lumbar spinal cord tissue at time points up to 48 hours after formalin injection. In Figure 2, 3, 4, 5, 6 the time courses of spinal cord expression of these genes are displayed. The components of the CO/NO-cGMP signaling system were grouped into HO isoforms (Figure 2), NOS isoforms (Figure 3), sGC isoforms (Figure 4), PKG isoforms (Figure 5), and PDE isoforms (Figure 6). Each of these groups had at least one member showing increased expression during the period of observation. In fact, 11/13 components had an increased mRNA level measured at one or more time points. There was no consistent temporal pattern for these increases in expression, though the final 48 hour time point was the most common one (8/13 genes) for increased expression to be observed while the 8 hour time point was the point least likely (0/13 genes) to be associated with increased expression. For only one gene was there observed to be a significantly decreased mRNA level at any time point (iNOS), and this was observed only at 8 hours post formalin injection. For no gene did saline injection significantly change expression at any time point analyzed (data not shown).

### Expression of components of the CO/NO-cGMP signaling system – protein

**Figure 7:**
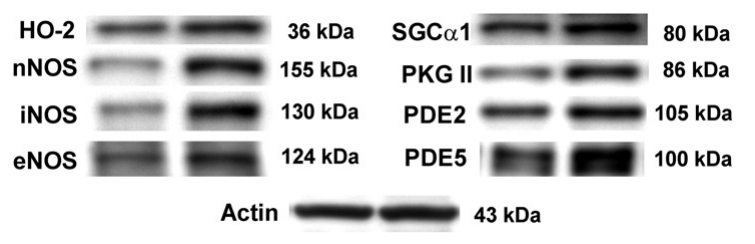
Immunoblots for selected CO/NO-cGMP signaling system proteins. Spinal cord protein samples from control animals (left hand lanes) and animals treated 48 hours previously with formalin (right hand lanes) are displayed for the 8 proteins found to display elevated expression at this time point. The molecular weights of the proteins are displayed next to the bands. After processing for CO/NO-cGMP signaling system proteins, blots were stripped and incubated with anti-actin antibodies in order to normalize for protein loading differences. The results of one such actin immunoblot is presented in the lower part of the figure.

With the mRNA measurements in hand we turned to the issue of whether the changes in message level corresponded to changes in actual protein content in spinal cord tissue. Table 2 provides a summary of the protein measurements, and Figure 7 shows the appearance of the bands observed on immunoblots. We chose for these studies the 2 hour time point because we desired to measure possible protein level changes in the acute period following nociceptive behaviors. We hypothesized that no protein level changes would be observed this close to the time of formalin injection. The 48 hour time point was chosen because our pervious studies had shown that spinal cord protein accumulation lagged behind mRNA level changes after hindpaw formalin stimulation [13], and our primary interest was in the changes in protein content that might support the persistent mechanical allodynia characteristic of this pain model. Of the 11 genes displaying an increase in mRNA at one or more time points, none displayed a greater protein level 2 hours after formalin injection. However, 8 were observed to have an increased spinal content of protein at 48 hours after formalin injection. For one protein (PDE3) we were not able to optimize our assay sufficiently to allow reliable detection despite the use of multiple antibodies and assay conditions. The 2 genes not having an increased mRNA level at any time point studied also had no increase in protein at 48 hours post formalin injection. The analysis of samples from morphine treated mice revealed that morphine treatment prior to formalin injection eliminated the changes in protein expression seen at 48 hours (Table 2).

**Table 2:** Summary of measured changes in spinal cord protein levels. The data presented represent the fold changes in spinal cord protein levels +/- SEM relative to control mice 2 and 48 hours after formalin injection. Some mice received 10 mg/kg morphine one time 30 min prior to formalin injection. *p < 0.05, **p < 0.01, ***p < 0.001. NRD, Not readily detected.

**Gene**	**Formalin (2 hr)**	**Formalin (48 hr)**	**Formalin (48 hr)+ Morphine**
HO-1	1.00 +/- 0.03	1.03 +/- 0.02	1.02 +/- 0.08
HO-2	1.01 +/- 0.08	1.14 +/- 0.02*	0.92 +/- 0.09
			
NOS-1	1.05 +/- 0.04	2.29 +/- 0.07***	0.84 +/- 0.13
NOS-2	1.06 +/- 0.06	2.00 +/- 0.11***	1.00 +/- 0.13
NOS-3	1.01 +/- 0.03	1.16 +/- 0.01**	1.05 +/- 0.07
			
sGCα1	1.00 +/- 0.04	1.24 +/- 0.06**	1.02 +/- 0.05
sGCα2	0.99 +/- 0.03	0.96 +/- 0.01	1.09 +/- 0.03
			
PKG Iα	0.99 +/- 0.02	1.00 +/- 0.01	1.04 +/- 0.02
PKG Iβ	1.00 +/- 0.05	1.03 +/- 0.01	0.99 +/- 0.02
PKG II	1.01 +/- 0.05	1.17 +/- 0.01*	0.99 +/- 0.08
			
PDE 2	0.99 +/- 0.04	1.16 +/- 0.01*	1.06 +/- 0.06
PDE 3	NRD	NRD	NRD
PDE 5	0.99 +/- 0.05	1.25 +/- 0.02**	1.00 +/- 0.08

## Discussion

The spinal cord CO/NO-cGMP system has received much attention, and is thought to regulate both nociceptive and analgesic pathways. The existing published studies are significantly limited, however. These limitations include, 1) the study of single molecules to infer roles for the much more complex enzymatic pathway, 2) the use of multiple species and multiple strains of individual species making the results of separate studies difficult to interpret together, and 3) the arbitrary lack of examination of many of this pathway's enzyme isoforms.

**Figure 8:**
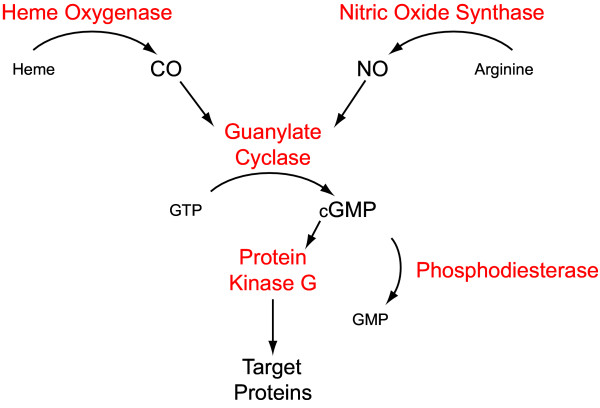
Diagrammatic representation of the CO/NO-cGMP signaling system.

In the present studies we attempted to include the majority of CO/NO-cGMP signaling system components in a time course study following the levels of spinal cord expression of corresponding genes in a commonly used model of inflammatory pain. Overall, at each CO/NO-cGMP system enzymatic level, we identified at least one gene having correspondingly elevated mRNA and protein levels after formalin injection. The principal observations of our studies were, 1) C57Bl/6J mice display not only robust phase I/II licking behaviour after formalin injection, but also a long lasting mechanical allodynia, 2) the majority of the CO/NO-cGMP genes selected for study had correspondingly elevated mRNA levels at some time point within 48 hours of formalin injection, 3) of the genes showing increased mRNA levels at some time point, most of those had correspondingly elevated protein levels as measured in spinal cord homogenates collected 48 hours after formalin injection, and 4) pre-treatment with the analgesic morphine eliminated the acute nociceptive response to formalin injection as well as the delayed changes in spinal cord protein expression, but not chronic nociceptive sensitisation. Figure 8 provides a diagram of the CO/NO-cGMP signaling system highlighting the principal enzymatic steps.

These observations of changes in expression do not by themselves constitute comprehensive evidence of participation in inflammatory pain for each gene. However, our observations do support the hypothesis that within the spinal cord multi-level changes in CO/NO-cGMP system gene expression follow noxious stimulation. Previously provided pharmacological evidence demonstrates the participation of HO [2,3], NOS [14,15], sGC [7] and PKG [8,9] in at least the acute phases of formalin stimulation. PDE inhibitors did not change acute formalin-induced pain behaviours in one study, though those investigations did not look at effects on persistent allodynia or changes in expression [10]. Also, HO-2 null mutants have reduced acute phase licking behaviour and long term allodynia after formalin injection [13], nNOS null mutants have normal formalin-induced licking but lack sensitivity to a NOS inhibitor, and PKG I null mutants have reduced formalin-induced licking behaviour [16]. Previous pharmacological and genetic studies provide valuable evidence concerning the functioning of the various individual components in support of nociceptive signaling. The present data, however, force us to consider the CO/NO-cGMP system as a functional unit with multiple components responding with some degree of coordination.

In the Background section several papers from the existing literature were cited which in various rodent pain models documented alterations in the expression of some of these genes. Though our results are largely consistent with the existing literature, there were differences between the present data and those provided in previous reports using similar paradigms. For example, 2 other groups have reported changes in PKG1α expression after hindpaw formalin injection where we found none [8,9]. Rather, the PKG II isoform seemed to have more robust changes in both mRNA and protein levels in our hands. The use of different species, rats versus mice, provides one possible explanation for the differences in observations. None-the-less, such differences highlight the importance of signaling system components being studied simultaneously if possible to eliminate model, strain and laboratory specific factors from confounding interpretation of the results as may occur when comparing results from the disparate existing literature.

Our morphine data were particularly helpful in understanding the mechanisms governing the control of expression of the CO/NO-cGMP system genes and the consequences of the enhanced expression. It was observed that the injection of a dose of morphine prior to formalin injection providing no more than 2 hours of analgesia in these mice [17] eliminated the wide-spread enhancement of expression of CO/NO-cGMP system genes. Thus it appears both that 1) the enhanced expression of CO/NO-cGMP system genes requires the intense acute nociception characteristic of formalin injection, and 2) that long term hind paw sensitisation does not require enhanced expression of these genes. This is not to say that the enzymatic activity of the existing proteins was not altered. Likewise, abundant pharmacological data indicates that some degree of activity of the CO/NO-cGMP system is required to support nociceptive sensitisation caused by formalin administration. Yet, it is clear that the simple observation of enhanced gene expression occurring in the context of increased pain sensitivity does not prove the enhanced expression is required for that increased sensitivity. In the formalin model the changes in gene expression might be considered indicators of the gene product's involvement in nociceptive signaling, not evidence for the necessity of an increase in gene expression in order for nociceptive sensitisation to occur. Also, though several of the genes studied are most prominently expressed in sensory processing areas of spinal cord dorsal horn tissue, e.g. HO-2 [3], more focused investigations looking at the precise spinal cord areas of altered gene expression may help us to more precisely define the function of those changes. The changes in gene expression we observed might be related to influencing processes other than nociception.

We should consider what new questions our observations raise. One set of issues is: By what mechanism is the response of the CO/NO-cGMP signaling system coordinated? For many years investigators have reported acute changes in spinal cord transcription factor levels after noxious formalin stimulation. Some of the identified factors include c-fos, Fos B, c-jun, jun B, jun D and CREB [18-20]. These could potentially distribute the nociceptive signal to multiple genes sharing particular transcription factor consensus sequences. It need to be recognized, however, that the time courses for mRNA expression increases were not identical for all genes we studies. Thus we would not necessarily expect a single mechanism or transcription factor to govern all the responses measured. In addition to transcription factors and CO/NO-cGMP signaling system components, many other types of molecules including ion channels, nociceptive neurotransmitters, enzymes and other molecules increase in spinal cord abundance after formalin injection [21-25]. We would hypothesize that ongoing investigation into the mechanistic basis for these changes will reveal the involvement of a relatively small group of signaling molecules and transcription factors which influence the expression of a large number of genes via interaction with common promoter region elements in the involved genes. Given the level of our knowledge of the murine genome and the available computational tools, comparison of promoter region sequences for genes responding in a similar manner to noxious stimuli like formalin is becoming feasible.

## Conclusion

Noxious inflammatory stimulation causes the increased expression of enzymes participating at each level in the CO/NO-cGMP signalling system. Changes in gene expression corresponding to components of this enzyme system are blocked by the co-administration of morphine though chronic nociceptive sensitisation can still occur. Future studies may be directed at understanding the coordinated CO/NO-cGMP signalling system response is achieved, and whether other pain-related enzyme systems share this type of multi-level response.

## Methods

### Animal use

All experimental protocols were reviewed and approved by the VAPAHCS institutional animal care and use committee prior to the initiation of work. Male mice 12–14 weeks old of the C57Bl/6J strain were kept under standard conditions with a 12 h light/dark cycle and allowed food and water ad libitum. Mice were obtained from Jackson Laboratories (Bar Harbor, MA) and were kept in our animal facility a minimum of 1 week prior to use in experiments.

### Drug administration

5% Formalin solution in 0.9% NaCl was made fresh on the day of experimentation. To induce inflammation, 20 μl of this solution was injected subcutaneously using a 27gauge needle and a microsyringe on the dorsal surface of both hind paws [19]. Control animals received injections of an equal volume of 0.9% NaCl. Animals also received subcutaneous injections of 0.9% NaCl or this vehicle containing 10 mg/kg morphine sulphate (Sigma Chemical, St. Louis, Mo) 30 min prior to formalin injection. Previous experiments demonstrated analgesia to be maximal within 30 minutes of morphine injection [17].

### Pain models

The formalin assay was carried out as we have previously described [2,13]. After the administration of formalin (see Drug Administration) the mouse was placed in a clear circular enclosure 25 cm in diameter with a glass floor, and the time spent licking the injected hind paw was measured. Phase I (0–5 min) hind paw licking was tabulated along with phase II licking (10–40 min) by recording total licking times in sequential 5 minute intervals.

Mechanical allodynia was assayed using nylon von Frey filaments according to the "up-down" algorithm described by Chaplan et al. [26] as we have used previously to detect allodynia after formalin injection [13]. In these experiments mice were placed on wire mesh platforms in clear cylindrical plastic enclosures of 10 cm diameter. After 20 minutes of acclimation, fibers of sequentially increasing stiffness (0.2–2 grams, 7 fibers) were applied to the centre of the plantar surface of the right hind paw just distal to the first set of foot pads and left in place 5 sec. Withdrawal of the hind paw from the fiber was scored as a response. When no response was obtained the next stiffest fiber in the series was applied to the same paw; if a response was obtained a less stiff fiber was next applied. Testing proceeded in this manner until 4 fibers had been applied after the first one causing a withdrawal response allowing the estimation of the mechanical withdrawal threshold [27].

### Tissue harvest for expression studies

Animals were sacrificed at specific time points by CO_2 _asphyxiation. Spinal cord lumbar segments (L3-S1) were harvested by extrusion and rapid dissection at the indicated times after formalin injection on a pre-chilled surface. Tissue was then quick frozen in liquid nitrogen and stored at -80°C until use.

### Total RNA isolation, reverse transcription and real-time PCR

The isolation of RNA and quantification using real time PCR were performed as described previously for spinal cord samples [12,13,28]. The isolation of total RNA was performed using the RNeasy Mini Kit (Qiagen, Valencia, CA) according to manufacturer's instructions. The purity and concentration was determined spectrophotometrically. Subsequently, cDNA was synthesized from this total RNA using random hexamer priming and a First Strand cDNA Synthesis Kit (Invitrogen, Carlsbad, CA). Briefly, 1 μg of total RNA was mixed with 4 μl of 10 × RT buffer, 8 μl of 25 mM MgCl2, 4 μl 0.1 M DTT, 1 μl RNasin, 2 μl SSII (50 u/μl), 5 μl hexomers and RNase-free water to 40 μl. Incubation was then carried out at 42°C for 60 minutes followed by heat inactivation at 70°C. Finally 1 μl RNase H was added to each reaction and incubated at 37°C for 20 minutes to degrade the RNA.

For real-time PCR, reactions were conducted in a volume of 4 μl using the Sybr Green I master kit (PE applied Biosystems, Foster City, CA). Briefly, 2 μl of a mixture of 2 × sybr green and primers (see Table 1) was loaded with 2 μl diluted cDNA template in each well. 8 μl mineral oil was loaded in each well to prevent loss of solution. PCR parameters were 95°C, 5 min then [95°C, 30 s→ 60°C, 30 s→ 72°C, 60 s] for 40 cycles. Melting curves were performed to document single product formation, and agarose electrophoresis confirmed product size. 18 s RNA was used as an internal control. The 18 s primers were purchased from Ambion (Austin, TX). Amplification kinetics for these products were found to be similar. The data from real-time PCR experiments were analysed by the comparative C_T _method. For these calculations average C_t _values from triplicate PCR reactions for CaMKIIα were normalized to average C_t _values for ones from 18 s from the same cDNA preparations. The ratio of comparative expression of each gene between the treated and untreated samples was calculated as 2^-(ΔΔCt)^. C_t _represents threshold cycle of PCR amplification. ΔC_t _represents the difference in threshold cycle between target and reference. ΔΔCt represents the difference between ΔC_t _(treatedsample) and ΔC_t _(untreated sample) for same gene.

**Table 1:** Summary of primer sequences and antibodies used. The sequence of forward (F) and reverse (R) primers used in PCR experiments are provided along with product sizes. Also provided are the sources for antibodies used and the dilution ranges.

**Gene**	**Primer Sequences**	**Product Size**	**Antibody Source, Cat#**	**Dilution Range**
HO-1	F:ACGCATATACCCGCTACCTG	227	Oncogene, PC340	1:100–1:500
	R:GAAGGCGGTCTTAGCCTCTT			
HO-2	F:ACTGAAGAAGGTTGCCCAGA	179	Santa Cruz, SC17786	1:100–1:500
	R:CTTTATTGGCCTCCTCCACA			
				
nNOS	F:TCAGTCTCCCAGGCTAATGG	200	Santa Cruz, SC5302	1:200–1:1000
	R:CTGTCCACCTGGATTCCTGT			
iNOS	F:CTCACTGGGACAGCACAGAA	199	Santa Cruz, SC650	1:200–1:500
	R:TGGTCAAACTCTTGGGGTTC			
eNOS	F:CTCACTGGGACAGCACAGAA	199	Santa Cruz, SC8311	1:200–1:1000
	R:TGGTCAAACTCTTGGGGTTC			
				
sGCα1	F:AGCGACTGAACCTTGCACTT	119	Sigma, G4280	1:1000–1:2000
	R:ACCTGCTGCAATTGCTTCTT			
sGCα2	F:CGAAAGCAACTTCGATGTGA	120	Santa Cruz, SC20954	1:100–1:400
	R:AAATGGGGTGGACAATCGTA			
				
PKG Iα	F:AAGCATGATGGGAAAACAGG	184	Santa Cruz SC10335	1:100–1:400
	R:GTGACTGCTGGCTTGTGGTA			
PKG Iβ	F:GACAGCTGCATCATCAAGGA	198	Stressgen KAP-PK002	1:500–1:1500
	R:GATGGCCCAGAGTTTCACAT			
PKG II	F:TGAACCGTGACGATGAAAAA	186	Santa Cruz SC25430	1:200–1:500
	R:CAAGCTCCACTCTTCCGAAC			
				
PDE 2	F:TTCAAGCTGCTGCAAGAAGA	224	Santa Cruz SC17227	1:200–1:1000
	R:TTCCTGAGGACCTGGATACG			
PDE 3	F:TTGCATAATTCAATGCCAAG	143	Multiple	Multiple
	R:TAGGTCCCGATCTTTTGCTG			
PDE 5	F:AAATGGTGGGACCTTCACTG	201	Cell Signaling 4072	1:200–1:1000
	R:GTGGCCGCTATCTTCTTCAG			

### Western blot analysis

We performed Western blot analysis for spinal cord samples as we have described previously [12,13,28,29]. Lumbar spinal cord tissue was homogenized in 56.8 mol/l Tris buffer, pH 6.8 with 1.8% (V/V) β-mercaptoethanol, 9.1% glycerol. The homogenate was centrifuged at 13,000 × g for 15 min at 4°C. The supernatant was decanted from the pellet and used for Western blot analyses. The concentration of protein in the homogenate was measured using DC Protein Assay kit (Bio-Rad, Hercules, CA). Equal amounts of protein (50 μg) were size fractionated by SDS-PAGE and electrotransferred onto a polyvinylidene difluoride membrane. The blots were blocked overnight with 5% non-fat dry milk in tris-buffered saline with 0.5% Tween-20 (TBST), incubated with primary antibody on a rocking platform at 4°C for 72 hrs. Primary polyclonal antibodies were obtained from the suppliers listed in Table 1 and used at the indicated dilutions. After washing in TBST, the blot was incubated 2 hrs at room temperature in horseradish peroxidase conjugated anti-rabbit or anti-goat antibody (diluted 1:2,000) (Chemicon, Temecula, CA), washed again, incubated in ECL Plus chemoluminescence reagents and exposed to Kodak XAY-2 film. Bands were quantified using scanning densitometry. Each blot was then stripped and re-probed with anti-β actin antibodies thus allowing normalization of expression between samples.

### Sample sizes

Experiments measuring acute and persistent pain related behaviors used 6 mice in each of the control, formalin and formalin plus morphine injected groups. For the quantification of mRNA levels, each time point represents the results from 12 mice with each resulting mRNA sample analyzed in triplicate or quadruplicate in each of at least 2 independent real time PCR experiments. The measurement of protein levels involved the use of 8 mice in each of the control, formalin and formalin plus morphine treated groups with homogenate samples analyzed in triplicate or quadruplicate on at least 2 Western blots.

### Statistical analysis

Analysis of repeated parametric measures was accomplished using an ANOVA analysis of variance for repeated measures followed by post-hoc t-testing. For simple comparisons of two means, two-tailed t-testing was performed. A value of p < 0.05 was taken to be significant. All data are presented as means +/- S.E.M. unless otherwise noted.

## Competing interests

The author(s) declare that they have no competing interests.

## Authors' contributions

XS performed the majority of the expression assays. This author was also responsible for design of the primer pairs used and the optimisation of PCR conditions.

XL performed several of the Western blotting experiments.

JDC was the senior investigator responsible for overall design, coordination and presentation of the experiments.
